# HiMSC and EV derived treatments increase Quality of Life and reduce amount of Knee Replacement Surgeries compared to current standard of care for knee osteoarthritis patients in The Netherlands

**DOI:** 10.1371/journal.pone.0344203

**Published:** 2026-03-26

**Authors:** Iris W. A. Boot, Georgina Shaw, Yolande F. M. Ramos, Mary Murphy, Ingrid Meulenbelt, Hubertus J. M. Vrijhoef, on behalf of the AutoCRAT consortium

**Affiliations:** 1 Panaxea b.v., Den Bosch, The Netherlands; 2 University of Galway, Galway, Ireland; 3 Leiden University Medical Center, Leiden, The Netherlands; Sheikh Hasina National Institute of Burn & Plastic Surgery, BANGLADESH

## Abstract

**Objectives:**

Current osteoarthritis treatments are designed to reduce pain and improve mobility instead of promoting the regeneration of cartilage. Cell-based therapies are being developed for the treatment of osteoarthritis. The aim of this study was to assess the cost-effectiveness of mesenchymal stromal cells and extracellular vesicle treatments compared to standard of care for patients with Kellgren-Lawrence stage II knee osteoarthritis in the Netherlands, from a hospital and societal perspective.

**Design:**

A Markov model was developed to assess the 40-year incremental cost-effectiveness ratio of mesenchymal stromal cells and/or extracellular vesicle treatments produced by automated or manual production methods, compared to standard of care. Secondary outcomes were amount of total knee replacement and total knee replacement revision surgeries. In addition, one-way sensitivity analyses and scenario analyses were performed.

**Results:**

The incremental cost-effectiveness ratios from a hospital perspective were -€10,982.10 for automatically produced mesenchymal stromal cells, -€9,301.79 for manually produced mesenchymal stromal cells, -€12,793.17 for automatically produced mesenchymal stromal cells and extracellular vesicle, and -€11,998.02 for manually produced mesenchymal stromal cells and extracellular vesicle versus standard of care. From the societal perspective incremental cost-effectiveness ratios were €68,870.58 for automatically produced mesenchymal stromal cells, -€67,280.27 for manually produced mesenchymal stromal cells, -€70,771.65 for automatically produced mesenchymal stromal cells and extracellular vesicle, and -€69,976.50 for manually produced mesenchymal stromal cells and extracellular vesicle versus standard of care. The amount of total knee replacement surgeries per 1,000 patients was 426 for the cell treatment groups, and 609 for the standard of care group, and 19 and 30 total knee replacement revision surgeries, respectively.

**Conclusions:**

This model, inherent to its assumptions, shows that hiMSC and EV treatments are cheaper and more effective for patients with knee osteoarthritis, from both perspectives. Moreover, they are expected to lower the number of surgeries. These conclusions persist among all scenario analyses.

## Introduction

Osteoarthritis (OA) is a leading cause of disability worldwide. OA is characterised primarily by the breakdown of articular cartilage, which results from degeneration of the extracellular matrix, resulting in pain, structural changes in the bone and joint space, and limitation of motion [1]. Individuals diagnosed with OA suffer physical weakness, possibly resulting in significant stress. Together, this leads to a diminished quality of daily life [1]. Disease onset is gradual and usually begins after the age of 40 [2]. The incidence of OA is rising due to the increase in life expectancy and the prevalence of obesity [3]. The number of people affected in Western Europe has grown 54% since 1990, with over 57 million people in 2019 living with OA [4]. In some countries, including The Netherlands, it is expected to become the most prevalent health condition as of 2040 [5].

Healthcare utilisation is extensive among knee OA patients, and this burden will increase with ageing populations [6]. Currently, both non-pharmacological and pharmacological methods are applied to treat OA. Non-pharmacological methods, including self-management, regular exercise, and weight control, are highly recommended and are regarded as first-line treatments for OA [1]. Pharmacological methods recommended in international guidelines are paracetamol and non-steroidal anti-inflammatory drugs (NSAIDs), which are often used when non-pharmacological methods are not able to relieve pain and reduce disability. Patients with hip and knee OA who do not respond to topical analgesics are recommended to take intra-articular corticosteroids [1]. Surgical options, such as joint replacement surgery, knee osteotomy, and knee joint distraction are recommended for patients with late-stage OA or young and energetic patients with moderate radiographic severity [1]. These treatments are designed to reduce pain and improve the mobility of joints instead of promoting the regeneration of damaged articular cartilage. Surgical interventions carry associated risks and expenses. It is argued by experts that surgical intervention should be postponed as long as possible, as having total knee replacement (TKR) at a younger age may increases the likelihood of requiring a revision, leading to increased costs and possible complications [7].

Although the specific pathogenesis of OA is variable, mesenchymal stromal cells (MSCs) and MSC-derived extracellular vesicles (EVs) represent a next-generation strategy for OA treatment, because of their anti-inflammatory and pro-reparative properties [1,7,8]. However, the cost of MSC and EV production prevents widespread use for patients. Manufacturing of MSCs and EVs at a scale suitable for therapeutic applications is complex and expensive. For instance, the process involves multiple stages including isolation, expansion, and purification, each requiring specialised equipment and stringent quality control to meet regulatory standards [9]. Besides, batch sizes are usually limited to the amount of starting material as MSCs are isolated from primary tissues [10].

The Horizon 2020 project Automated Cellular Robot-Assisted Technologies for translation of discovery-led research in Osteoarthritis (AutoCRAT) (grant agreement No. 874671) proposed the development of affordable and sustainable cell and cell-based therapies for OA with the concurrent development of closed, scalable, and regulatory-compliant automated systems for their manufacture to provide a complete solution for delivery of OA therapeutics to patients. Although MSCs from primary tissues have been successfully applied in the clinic, their expansion capabilities are limited and results are variable. MSCs derived from human-induced pluripotent stem cells (hiMSCs) are expected to overcome these limitations and serve as a reproducible and sustainable cell source [11]. As part of AutoCRAT an early health technology assessment was performed. This evaluation method is increasingly being used to support health economic evidence development during early stages of clinical research [12].

The aim of the current study is to assess the cost-effectiveness of AutoCRAT´s hiMSC and derived EV treatments compared to standard care for patients with Kellgren-Lawrence (KL) stage II knee OA in the Netherlands over a period of 40 years, from a hospital and societal perspective. The findings are expected to inform industry and other stakeholders about the potential value of this new medical product in development, including methods to quantify and manage uncertainty.

## Methods

### Study design and study population

To assess the cost-effectiveness hiMSC and EV treatments compared to the standard of care, a Markov-simulation model was developed [13].

Since not all patients are aware they have OA in KL stage I [14], and are therefore not likely to seek treatment, and because cell therapies are most effective when administered when the cartilage has not degenerated too far, it was decided to consider patients with KL stage II knee OA as the study population. Moreover, the study of Herbolsheimer et al. [15] has shown that there are large national variations between persons with knee OA, not only explained by disease-specific factors, but also social, environmental and other contextual factors that should be taken into account. Therefore, this study focuses on one country. Because of the expected availability of data to populate the Markov-simulation model, it was decided to focus on the Netherlands.

### Model overview

**Fig 1:**
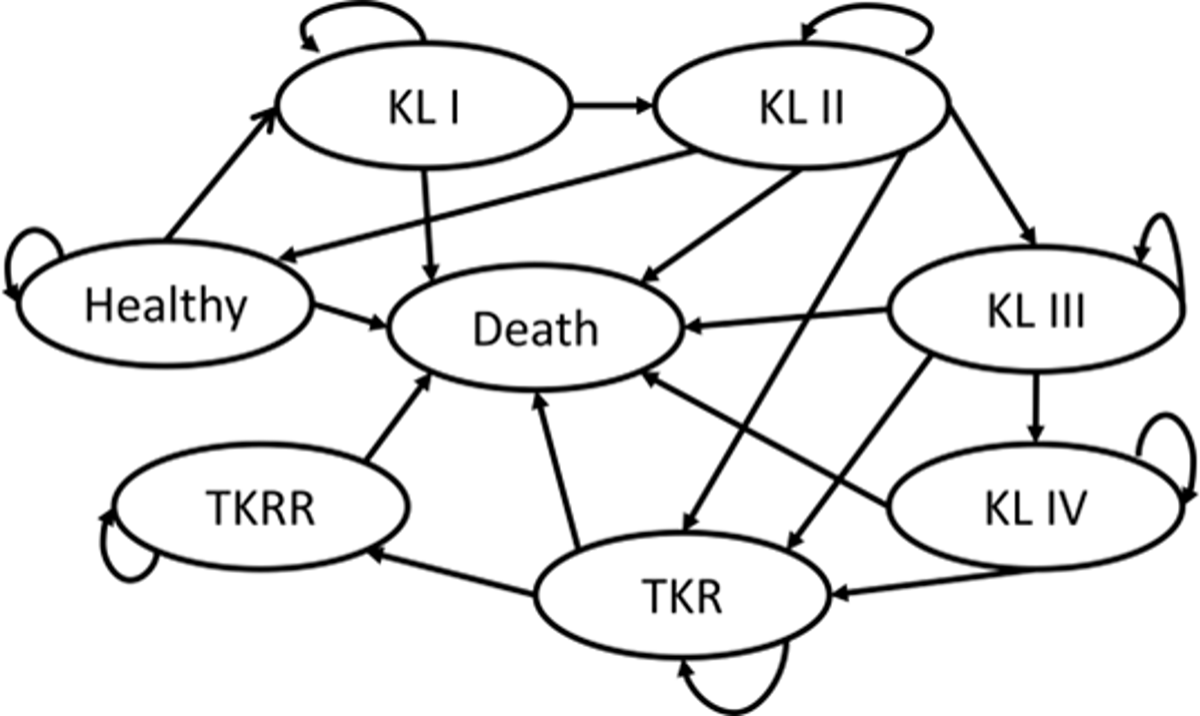
Markov model health states. Abbreviations: KL: Kellgren-Lawrence; TKR: total knee replacement; TKRR: total knee replacement revision.

The model used in this study simulates eight health states, including healthy, KL stage I, KL stage II, KL stage III, KL stage IV [14], total knee replacement (TKR), TKR revision (TKRR) and death, see Fig 1. Data were obtained from published literature and the AutoCRAT project to compare health state costs and quality adjusted life years (QALYs) between hiMSC treatment, hiMSC and EV treatment and treatment targeted at symptom reduction (i.e., standard of care).

The primary outcome of the analyses is the incremental cost-effectiveness ratio (ICER), which is calculated by dividing the difference in total costs (incremental cost) by the difference in QALYs (incremental effect) [16]. Secondary outcomes include the numbers for TKR and TKRR operations. Outcomes were discounted according to Dutch guidelines (i.e., 4% for costs and 1.5% for health outcomes) [17]. All analyses were performed from both the hospital and societal perspective, to be relevant for decision makers at different levels. The societal perspective includes key indirect cost components, primarily productivity losses due to patient absenteeism and caregiver time, which largely drive the observed differences in total costs [18].

Patients enter the model at KL stage II and receive either usual care, including physical therapy, medications such as acetaminophen, nonsteroidal anti-inflammatory drugs and glucosamine, imaging and aids such as knee braces and elastic bandage [19], or a hiMSC or EV treatment. They enter the model at 45 years old, a cycle length is one year, and there are 40 cycles in total. Data on health state utilities are derived from the Cohort Hip Cohort Knee (CHECK) study and literature [20,21]. Transition probabilities from KL health states are based on the CHECK study (2002–2005) [20]. The probability of TKRR is based on the Dutch Arthroplasty Registry [21] (See Table 1 in S1 Appendix). For standard care, people can only progress to a worse health state (i.e., from KL stage II to KL stage III, from KL stage III to KL stage IV, from any stage to TKR, from TKR to TKRR) or die (i.e., any state to death). Transition probabilities for the hiMSC and hiMSC and EV arms are similar to those of standard of care, except that the patients who receive a hiMSC or EV treatment, are assumed to receive treatment in KL stage II. For these patients, 69% of the treatments will be effective (i.e., regenerate cartilage) and, therefore, transition to the healthy state [22]. Given that evidence on hiMSC-derived EV effectiveness is currently restricted to a rat model [23], the base case analysis assumed comparable effectiveness between hiMSC and EV treatment as a working hypothesis. This assumption is biologically motivated by the central role of EV-mediated paracrine signalling in MSC therapeutic effects [24], however, remains provisional. Similarly, hiMSCs and hiMSC-derived EVs were assumed to be as effective as primary MSCs, despite limited direct comparative evidence. In the base case analysis, anyone who enters KL stage II receives a hiMSC or EV treatment (i.e., the treatment can be given multiple times to the same patients). Repeated administration is assumed in the base case analysis to explore the theoretical maximum health benefit of hiMSC and EV therapies under unrestricted access. All patients, irrespective of their treatment arm, are assumed to decline one health state per cycle at maximum. If there was a probability of declining two health states based on the CHECK data [20], this probability was added to the one state decline (e.g., if the probability of declining from state KL I to KL II was 0.1 and from KL I to KL III was 0.05 based on the data derived from the CHECK study [20], the probability of declining used in the model was (0.1 + 0.05=) 0.15 from KL I to KL II and 0 from KL I to KL III), see Table 2.

Risk of death was based on the Dutch registry (i.e., background mortality [19], see Table 2 in S1 Appendix) and risk associated with drug use [8] and surgery [25].

Costs per KL health state are based on a Dutch study from Hermans et al. [26]. Surgery costs and costs of TKR and TKRR states after the years of surgery are derived from the study of Van der Woude et al. [21]. Costs of the hiMSC and EV treatments were based on studies from the AutoCRAT project. Data on manufacturing processes, equipment and disposables used were retrieved from the Centre for Cell Manufacturing Ireland (CCMI) at the University of Galway. For these calculations, 330 manufacturing days were assumed for the automated process and 250 for the manual process, resulting in the production of 750 hiMSC and 480 hiMSC treatment units, and 750 hiMSC plus 750 EV and 480 hiMSC plus 480 EV treatment units, respectively, see Table 1-5 in S2 and S3 Appendix. Costs associated with death are derived from a study looking at the additional healthcare consumption of OA patients in their last year of life compared to the healthy population multiplied by Dutch unit prices [27,28]. All costs are indexed to 2024 prices using the price indices of the Dutch Healthcare Authority (see Table 1 in S4 Appendix) [29].

The model is constructed using the open-source software AMUA (version 3.0) Table 1.

**Table 1:** Baseline parameters.

	Hospital perspective			Societal perspective			
	Base case	Lower bound	Upper bound	Base case	Lower bound	Upper bound	
***Costs (2024 €/year)***							
Healthy	0	0	0	0	0	0	Assumption
KL I	1,927.30	931.31	2,561.11	11,266.30	1,384.03	15,521.88	
KL II	2,569.73	1,241.75	3,414.81	15,021.73	1,845.38	20,695.84	(24)
KL III	3,212.17	1,522.19	4,268.52	18,777.16	2,306.72	25,869.80	
TKR operation	16,565.05	11,602.44	22,398.71	16,565.05	11,602.44	22,398.71	(19)
TKR	0	0	0	0	0	0	(19)
TKRR operation	27,608.42	22,463.59	33,276.43	27,608.42	22,463.59	33,276.43	(19)
TKRR	1,380.42	519.04	2,654.55	1,380.42	519.04	2,654.55	(19)
hiMSC treatment automated	1,539.00*			1,539.00*			AutoCRAT
• *Facility*	*156,864.00*	*125,491.20*	*188,236.80*	*156,864.00*	*125,491.20*	*188,236.80*	
• *Staffing*	*421,814.00*	*337,451.20*	*506,176.80*	*421,814.00*	*337,451.20*	*506,176.80*	
• *Equipment depreciation*	*136,697.00*	*109,357.60*	*164,036.40*	*136,697.00*	*109,357.60*	*164,036.40*	
• *Consumables*	*249,238.00*	*199,390.40*	*299,085.60*	*249,238.00*	*199,390.40*	*299,085.60*	
• *Quality control*	*190,000.00*	*152,000.00*	*228,000.00*	*190,000.00*	*152,000.00*	*228,000.00*	
• *MSC units per year, automated*	*750*	*600*	*900*	*750*	*600*	*900*	
hiMSC and EV treatments automated	770.00 ^a^			770.00 ^a^			AutoCRAT
• *hiMSC and EV units per year, automated*	*1,500*	*1,200*	*1,800*	*1,500*	*1,200*	*1,800*	
hiMSC treatment manual	2,183.00^b^			2,183.00 ^b^			AutoCRAT
• *Facility*	*231,775.00*	*185,420.00*	*278,130.00*	*231,775.00*	*185,420.00*	*278,130.00*	
• *Staffing*	*454,955*	*363,964.00*	*545,946.00*	*454,955*	*363,964.00*	*545,946.00*	
• *Equipment depreciation*	*49,646.00*	*39,716.80*	*59,575.20*	*49,646.00*	*39,716.80*	*59,575.20*	
• *Consumables*	*176,656.00*	*141,324.80*	*211,987.20*	*176,656.00*	*141,324.80*	*211,987.20*	
• *Quality control*	*135,000.00*	*108,000.00*	*162,000.00*	*135,000.00*	*108,000.00*	*162,000.00*	
• *MSC units per year, automated*	*480*	*384*	*576*	*480*	*384*	*576*	
hiMSC and EV treatments manual	1,092.00**			1,092.00**			AutoCRAT
• *hiMSC and EV units per year, automated*	*960*	*768*	*1,800*	*960*	*768*	*1,152*	
Death	1,082.28	1,163.91	1,745.87	1,082.28	865.83	1,298.74	(25, 26)
***Utility score***							
Healthy	1.00	1.00	1.00	1.00	1.00	1.00	Assumption
KL I	0.80	0.64	0.96	0.80	0.64	0.96	(18)
KL II	0.82	0.66	0.98	0.82	0.66	0.98	(18)
KL III	0.77	0.62	0.92	0.77	0.62	0.92	(18)
TKR	0.79	0.76	0.82	0.79	0.76	0.82	(19)
TKR operation cycle	0.76	0.73	0.79	0.76	0.73	0.79	(19)
TKRR	0.75	0.72	0.78	0.75	0.72	0.78	(19)
TKRR operation cycle	0.73	0.70	0.76	0.73	0.70	0.76	(19)

Abbreviations: KL: Kellgren-Lawrence; TKR: total knee replacement; TKRR: total knee replacement revision; AutoCRAT: Automated Cellular Robot-Assisted Technologies; CHECK: Cohort Hip and Cohort Knee; hiMSC: human induced mesenchymal stromal cells; EV: extracellular vesicles.

^a^: see Table 1-5 in S2 Appendix for detailed calculations.

^b^: see Table 1-5 in S3 Appendix for detailed calculations.

### Sensitivity analyses

One-way sensitivity analyses are conducted to determine the main cost drivers. The lower and upper values of the ranges used in the analyses correspond to the 95% confidence intervals reported by a single study. If neither was available, the value was increased and decreased by 20% [30].

### Scenario analyses

A first scenario analysis was performed in which people can only receive a hiMSC or EV treatment once. Due to the scarcity of financial resources, and consistency with current clinical, regulatory, and reimbursement expectations for cell-based therapies, this scenario was considered of high relevance for decision makers and is therefore presented.

A second scenario analysis was performed in which the probability of getting knee OA (i.e., transition probability from Healthy state to KL I state) was based on the Dutch information of Public Health and Care (p = 0.04345) [31]. The base case transition probability from Healthy to KL I was based on the CHECK study [20], which consists of people who already have knee complaints. If a hiMSC or EV treatment cures people to healthy, then the general Dutch population might be a better representation of the transition probability from the Healthy state to state KL I.

A third scenario analysis was performed in which the effect of hiMSC and EV treatments are derived from the results of the AutoCRAT project. Herein, transition probabilities from KL II to Healthy state were 0.76 for all hiMSC and EV treatments which was determined by intra-articular human induced pluripotent stem cell-derived MSCs and EVs loaded in thermos-sensitive hydrogel in a surgical destabilization of the medial meniscus mouse model of osteoarthritis [32] (study conducted at the Leiden University Medical Centre, approved by Animal Welfare Committee (IvD) under number AVD1160020171405-PE.18.101.006 on April 21, 2023) Table 2.

**Table 2:** Transition probabilities base-case.

	Healthy	KL I	KL II	KL III	KL IV	TKR	TKRR	Death
Healthy	0.5398	0.5165	0	0	0	0	0	^b^
KL I	0	0.7407	0.2626	0	0	0.0046	0	^b^
KL II	0/0.69^a^	0	0.928	0.0291	0	0.0092	0	^c^
KL III	0	0	0	0.8598	0	0.0864	0	^c^
KL IV	0	0	0	0	0.7	0.3	0	^c^
TKR	0	0	0	0	0	1-X	X	^d^
TKRR	0	0	0	0	0	0	1	^d^
Death	0	0	0	0	0	0	0	1

*Transition probabilities from each health state should add up to 1. Therefore, in the analyses, each transition probability is corrected proportionally by multiplying the value by one divided by the sum of transition probability values for that cycle. This sum varies per cycle.*

Abbreviations: KL: Kellgren-Lawrence; TKR: total knee replacement; TKRR: total knee replacement revision.

^a^: 0.69 for hiMSC or EV treatment, 0 for standard of care.

^b^: background mortality (see Table 2 in S1 Appendix).

^c^: background mortality (see Table 2 in S1 Appendix) and probability of dying due to use of OA drugs (p = 0.0012) [10].

^d^: background mortality (see Table 2 in S1 Appendix) and probability of dying due to operation (p = 0.002) [24] (only in first cycle, other cycles only include background mortality).

^X^: probability of having TKRR after TKR. Differs per year after TKR surgery (see Table 1 in S1 Appendix).

Since the base case analysis is likely optimistic due to the assumption of regressing to the Healthy state after receiving a hiMSC or EV treatment, a fourth and last scenario analysis was performed in which people receiving a hiMSC or EV treatment return to state KL I. This scenario analysis was based on observations from the AutoCRAT partner Leiden University Medical Center (LUMC) on human cartilage samples.

### Ethics

In the Netherlands, ethical approval for research is required if the study falls under the scope of the Dutch Medical Research involving Human Subjects Act (In Dutch: Wet medisch-wetenschappelijk onderzoek met mensen, WMO) [33,34]. Research is subjected to this Act if it meets two criteria: 1) The research qualifies as medical-scientific research, and 2) it involves subjecting participants to procedures or imposing specific rules of behaviour on them. For this study, no new human data were collected and all analyses were based on existing datasets and modelling inputs, including animal data for scenario analysis.

As our study did not meet these criteria, approval was not needed according to Article 1b of the Dutch Medical Research in Human Subjects Act [34].

## Results

### Primary results

This study showed that hiMSC and EV treatments, irrespective of how they are produced, are expected to reduce the treatment costs per patient and increase the QALYs (i.e., cheaper and more effective) for patients with knee OA KL stage II in the Netherlands, from both a hospital and societal perspective, over a period of 40 years, irrespective of how they were produced. This is shown in the ICERs, see Table 3.

**Table 3:** Overview of primary and secondary results in the base case analysis.

		Hospital perspective		Societal perspective			
	QALYs per person over 40 years	Costs per person over 40 years (€)	ICER (compared to SoC) (€)	Costs per person over 40 years (€)	ICER (compared to SoC) (€)	TKR	TKRR
hiMSC automated	22.796	35,569.25	−10,982.10	175,023.42	−68,870.58	426	19
hiMSC manual	22.796	36,870.66	−9,301.79	176,324.82	−67,280.27	426	19
hiMSC and EV automated	22.796	34,013.54	−12,793.17	173,467.71	−70,771.65	426	19
hiMSC and EV manual	22.796	34,664.25	−11,998.02	174,118.41	−69,976.50	426	19
Standard of care	21.978	44,482.61		231,382.41		609	30

Abbreviations: QALY: quality adjusted life year; ICER: incremental cost-effectiveness ratio; TKR: total knee replacement; TKRR: total knee replacement revision; hiMSC: human induced mesenchymal stromal cells; EV: extracellular vesicles.

### Secondary results

The amount of TKR surgeries performed per 1,000 patients over 40 years was 426 for the hiMSC and EV treatment groups, and 609 for the standard of care group (Table 3). This represents a 30.0% difference of TKR surgeries in favour of hiMSC and EV treatments.

The amount of TKRR surgeries performed per 1,000 patients over 40 years was 19 for the hiMSC and EV treatment groups, and 30 for the standard of care group (Table 3). This represents a 36.7% difference in TKRR surgeries in favour of hiMSC and EV treatments.

### Sensitivity analyses

An overview of the model input parameters with the largest impact on the costs for hiMSC treatment arm can be found in Table 4. Here we see that the yearly health state costs of health state KL I, II and III and the effectiveness of the hiMSC treatment have the largest impact for all perspectives and production processes.

**Table 4:** Top 10 model input parameters that are the main cost drivers for hiMSC treatments (1 = most impact).

	Automatically produced		Manually produced	
	Hospital	Societal	Hospital	Societal
1	c_KLII	c_KLII	c_KLII	c_KLII
2	p_KLII_Healthy_MSC	c_KLI	p_KLII_Healthy_MSC	c_KLI
3	c_KLI	p_KLII_Healthy_MSC	c_KLI	p_KLII_Healthy_MSC
4	c_KLIII	c_KLIII	c_KLIII	c_KLIII
5	c_TKR_operation	redo_MSCTx	c_TKR_operation	redo_MSCTx
6	redo_MSCTx	p_KLII_KLII	MSC_units_year_manual	p_KLII_KLII
7	p_Healthy_KLI	p_Healthy_KLI	p_Healthy_KLI	p_Healthy_KLI
8	MSC_units_year_automated	p_KLIII_TKR	redo_MSCTx	p_KLIII_TKR
9	p_Healthy_Healthy	p_KLIII_KLIII	p_Healthy_Healthy	p_KLIII_KLIII
10	p_KLII_KLII	p_Healthy_Healthy	p_KLII_KLII	p_Healthy_Healthy

Abbreviations: c_KLII: yearly costs of health state Kellgren-Lawrence II; p_KLII_Healthy_MSC: probability of regressing from KL state II to health state Healthy after receiving hiMSC treatment c_KLI: yearly costs of health state KL I; c_KLIII: yearly costs of health state KL III; c_TKR_operation: costs of TKR procedure; redo_MSCTx: possibility of receiving hiMSC treatment again when entering health state KL II; p_Healthy_KLI: probability of progressing to state KL I from health state Healthy; MSC_units_year_automated: the number of treatment units that can be produced per year; p_Healthy_Healthy: probability of staying in health state Healthy; p_KLII_KLII: probability of staying in health state KL II; p_KLIII_TKR: probability of undergoing TKR from health state KL III; p_KLIII_KLIII: probability of staying in health state KL III; MSC_units_year_manual: the number of treatment units that can be produced per year; KL: Kellgren-Lawrence; hiMSC: human induced mesenchymal stromal cells; TKR: total knee replacement; OA: osteoarthritis.

An overview of the results of the sensitivity analyses from hiMSC and EV treatments can be found in Table 5. Irrespective of the perspective and production process, Table 5 shows the input parameters that are the main cost drivers are the costs of health state KL I, II and III, and the effectiveness of the hiMSC and EV treatments.

**Table 5:** Top 10 model input parameters that are the main cost drivers for hiMSC and EV treatments (1 = most impact).

	Automatically produced		Manually produced	
	Hospital	Societal	Hospital	Societal
1	c_KLII	c_KLII	c_KLII	c_KLII
2	p_KLII_Healthy_MSC	c_KLI	p_KLII_Healthy_MSC	c_KLI
3	c_KLI	p_KLII_Healthy_MSC	c_KLI	p_KLII_Healthy_MSC
4	c_KLIII	c_KLIII	c_KLIII	c_KLIII
5	c_TKR_operation	redo_MSCTx	c_TKR_operation	redo_MSCTx
6	redo_MSCTx	p_KLII_KLII	redo_MSCTx	p_KLII_KLII
7	p_Healthy_KLI	p_Healthy_KLI	p_Healthy_KLI	p_Healthy_KLI
8	p_Healthy_Healthy	p_KLIII_TKR	p_Healthy_Healthy	p_KLIII_TKR
9	p_KLII_KLII	p_KLIII_KLIII	EV_units_year_manual	p_KLIII_KLIII
10	p_KLIII_KLIII	p_Healthy_Healthy	p_KLII_KLII	p_Healthy_Healthy

Abbreviations: c_KLII: yearly costs of health state Kellgren-Lawrence II; p_KLII_Healthy_MSC: probability of regressing from KL state II to health state Healthy after receiving EV treatment; c_KLI: yearly costs of health state KL I; c_KLIII: yearly costs of health state KL III; c_TKR_operation: costs of TKR procedure; redo_MSCTx: possibility of receiving EV treatment again when entering health state KL II; p_Healthy_KLI: probability of progressing to state KL I from health state Healthy; p_Healthy_Healthy: probability of staying in health state Healthy; p_KLII_KLII: probability of staying in health state KL II; p_KLIII_KLIII: probability of staying in health state KL III; p_KLIII_TKR: probability of undergoing TKR from health state KL III; EV_units_year_manual: the number of treatment units that can be produced per year; KL: Kellgren-Lawrence; hiMSC: human induced mesenchymal stromal cells; EV: extracellular vesicles; TKR: total knee replacement; OA: osteoarthritis.

An overview of the results of the sensitivity analyses from standard of care can be found in Table 6. Here it becomes apparent that the costs of a TKR surgery have a greater impact for standard of care than for hiMSC and EV treatments. Also, transition probabilities have a greater impact.

**Table 6:** Top 10 model input parameters that are the main cost drivers for standard of care (1 = most impact).

	Hospital	Societal
1	c_KLII	c_KLII
2	c_KLIII	c_KLIII
3	c_TKR_operation	p_KLII_KLII
4	p_KLII_KLII	p_KLIII_TKR
5	p_KLIII_KLIII	p_KLIII_KLIII
6	p_KLIII_TKR	p_KLII_TKR
7	p_KLII_TKR	p_KLII_KLIII
8	c_TKR	c_TKR_operation
9	p_KLII_KLIII	p_mortalityOAdrugs
10	c_TKRR	c_TKR

Abbreviations: c_KLII: yearly costs of health state Kellgren-Lawrence II; c_KLIII: yearly costs of health state KL III; p_KLII_KLII: probability of staying in health state KL II; p_KLIII_TKR: probability of undergoing TKR from health state KL III; p_KLIII_KLIII: probability of staying in health state KL III; p_KLII_TKR: probability of undergoing TKR from health state KL II; p_KLII_KLIII: probability of progressing from health state KL II to health state KL III; c_TKR_operation: costs of TKR procedure; p_mortalityOAdrugs: probability of dying due to the use of OA drugs; c_TKR: yearly costs of health state TKR; c_TKRR: yearly costs of health state TKRR; KL: Kellgren-Lawrence; EV: extracellular vesicles; TKR: total knee replacement; OA: osteoarthritis.

Detailed results, including tornado diagrams and expected costs per patient over 40 years for each of the top 10 model input parameters that are the main cost drivers for a treatment are presented in Figs 1-10 and Table 1-10 in S5 Appendix.

### Scenario analyses

All scenario analyses performed showed that hiMSC and EV treatments, irrespective of how they are produced and the perspective taken in the analysis, are expected to reduce the treatment costs per patient and increase the QALYs (i.e., cheaper and more effective), see Table 7. In the first scenario analysis patients could receive a hiMSC or EV treatment once, in the second scenario analysis the transition probability from Healthy state to KL I state was lowered (i.e., p = 0.04345), in the third scenario analysis the effect of hiMSC and EV treatments were derived from the results of the AutoCRAT small-animal studies (i.e., 0.76), and in the fourth and final scenario analysis people receiving a hiMSC or EV treatment regress to state KL I instead of the Healthy state.

**Table 7:** Overview of primary and secondary results of the scenario analyses.

		Hospital perspective		Societal perspective			
	QALYs per person over 40 years	Costs per person over 40 years (€)	ICER (compared to SoC) (€)	Costs per person over 40 years (€)	ICER (compared to SoC) (€)	TKR	TKRR
*Scenario 1: Patients can only receive hiMSC or EV treatment once*							
hiMSC automated	22.337	40,359.08	−11,477.80	205,271.47	−72,679.50	546	25
hiMSC manual	22.337	41,002.99	−9,685.46	205,915.38	−70,887.17	546	25
hiMSC and EV automated	22.337	39,589.34	−13,620.36	204,501.72	−74,822.07	546	25
hiMSC and EV manual	22.337	39,911.29	−12,724.20	204,823.68	−73,925.90	546	25
Standard of care	21.978	44,482.61		231,382.41		609	30
*Scenario 2: Lower transition probability from Healthy state to KL I state*							
hiMSC automated	23.304	29,991.17	−10,931.02	146,811.90	−63,792.31	358	16
hiMSC manual	23.304	31,128.01	−10,073.49	147,948.74	−62,934.78	358	16
hiMSC and EV automated	23.304	28,632.18	−11,956.12	145,452.91	−64,817.40	358	16
hiMSC and EV manual	23.304	29,200.60	−11,527.36	146,021.33	−64,388.64	358	16
Standard of care	21.978	44,482.61		231,382.41		609	30
*Scenario 3: Effect of hiMSC and EV treatments are derived from the results of the AutoCRAT small-animal studies*							
hiMSC automated	22.990	33,761.80	−10,593.69	165,070.04	−63,568.71	375	16
hiMSC manual	22.990	35,199.61	−9,172.92	166,507.85	−64,105.30	375	16
hiMSC and EV automated	22.990	32,043.03	−11,912.12	163,351.27	67,224.45	375	16
hiMSC and EV manual	22.990	32,761.93	−11,581.70	164,070.18	−66,514.06	375	16
Standard of care	21.978	44,482.61		231,382.41		609	30
*Scenario 4: People receiving a hiMSC or EV treatment do not regress to the Healthy state, but to state KL I*							
hiMSC automated	22.113	40,918.18	−26,432.06	202,074.46	−217,333.54	477	21
hiMSC manual	22.113	42,368.13	−15.679.91	203,524.41	−206,581.39	477	21
hiMSC and EV automated	22.113	39,184.90	−39,285.25	200,341.17	−230,186.73	477	21
hiMSC and EV manual	22.113	39,909.87	−33,909.18	201,066.15	−244,810.66	477	21
Standard of care	21.978	44,482.61		231,382.41		609	30

Abbreviations: QALY: quality adjusted life year; ICER: incremental cost-effectiveness ratio; TKR: total knee replacement; TKRR: total knee replacement revision; hiMSC: human induced mesenchymal stromal cells; EV: extracellular vesicles; KL: Kellgren-Lawrence.

## Discussion

In this study, based on its assumptions, hiMSC and EV treatments produced by the AutoCRAT production process have been shown to be dominant over standard care for patients with KL stage II knee OA in the Netherlands over a period of 40 years from both a hospital and societal perspective. In light of the cost-effectiveness threshold for treatments that target diseases with the lowest burden of illness in the Netherlands, in which OA falls [35], the ICERs for hiMSC and EV treatments compared to standard of care are below the threshold value (i.e., a maximum cost of €20,000 per QALY gained). This would imply that the novel treatments could be considered for reimbursement.

Besides the favourable incremental cost-effectiveness, the health economic model also showed a decrease in TKR and TKRR surgeries, in the base case analysis and in the scenario analyses performed. Since the base case analysis is likely optimistic, it is important to consider the scenario analysis evaluating the impact of the assumptions on the results. In The Netherlands, 26,707 TKR surgeries were performed in 2022 [36]. With an ageing population, this number is expected to rise to 57,000 in 2030 [35]. Even if it is acceptable to treat patients only once with a hiMSC or EV treatment and therefore not be completely curative, it is essential to prolong the time between the decline of conservative treatment efficacy and surgical intervention, as having TKR at a younger age may increase the likelihood of requiring a revision, leading to increased costs and possible complications [7]. Therefore, it is important to consider treatment options available that may alleviate symptoms related to knee OA and delay the need for TKR to reduce the likelihood of requiring a TKRR [7].

Results of this study can be used in the discussion with Dutch stakeholders, including investors and policymakers, regarding the incremental cost-effectiveness of hiMSC and EV treatments and reimbursement decisions. As cell-based therapies are likely to be administered once, due to current clinical, regulatory, and reimbursement expectations, the single-treatment scenario is particularly relevant for policymakers. The base-case analysis provides a theoretical construct exploring the maximum health benefit of hiMSC and EV therapies. Moreover, the findings can be used as an example in other countries with comparable healthcare systems.

### Strengths and limitations

To our knowledge, this study is the first to assess the cost-effectiveness of hiMSC and EV treatments for knee OA in the Dutch healthcare setting from both a hospital and societal perspective. A strength of the current study is the collection of Dutch unit cost data to explore the potential impact of the treatment in the Dutch healthcare setting. Besides, this study provides a model that can be updated when new data becomes available. However, the study has several limitations. First, because the new technology has not been implemented yet, results should be interpreted with caution. The analyses in the current study rely on data from the AutoCRAT project and existing literature, these should be updated ones more empirical data become available. Since the follow-up of the studies included was no longer than twelve months, assumptions were required to extrapolate treatment effects over the 40-year time horizon used to reflect the chronic nature of osteoarthritis. This long-term extrapolation introduces uncertainty, particularly given the early developmental stage of the evaluated therapies and the possibility that treatment effects may wane over time. These assumptions may influence the estimated cost-effectiveness outcomes, underscoring the importance of updating efficacy inputs and exploring alternative time horizons or effect-duration scenarios when longitudinal data become available.

Additionally, it was not possible to collect data on all production costs for the Dutch setting. Therefore, data from the CCMI were used. In general, Ireland has higher laboratory production costs, which leads to higher costs per cell treatment unit. This would imply that the costs used in this study are an overestimation of the actual costs in the Netherlands, and therefore the overall results are regarded as conservative estimates.

Although results generated in the AutoCRAT project are preferred to estimate the effect of the hiMSC and EV treatments in the base case analysis, these were included as a scenario analysis. Unfortunately, due to the small number of large-animal study results (i.e., n = 6), no robust estimate of the effect of hiMSC and EV treatments only was available. Hereto the results of intra-articular injections of hiMSC and EV therapies in a surgical destabilization of the medial meniscus mouse model of osteoarthritis were used (unpublished data Ramos and Meulenbelt).

In this study, no patients with KL stage IV OA were included because there were no data available in the CHECK database for these patients [17]. However, when data for these patients become available in the future, the incremental cost-effectiveness of cell treatments could also be assessed.

Despite these limitations, early HTAs are highly valuable because they provide critical insights into the potential clinical and economic impact of this new medical product. They support informed decision-making in early development stages, helping prioritize investments, guide research efforts, and identify key data gaps to address before full-scale implementation [37].

## Conclusions

The objective of this study was to assess the incremental cost-effectiveness of AutoCRAT hiMSC and EV treatments compared to standard care for patients with KL stage II knee OA in the Netherlands over a period of 40 years from a hospital and societal perspective. Based on the available evidence, the model-based analysis showed that both the hiMSC and EV treatments, either automatically or manually produced, might lead to an increase in QALYs, a decrease in costs per patient and a decrease in amount of knee replacement surgeries. This was the case for the base case analysis and various performed scenario analyses.

## Supporting information

S1 Appendix Transition probabilities. (DOCX)

S2 Appendix Costs related to automated hiMSC and EV treatment manufacturing process. (DOCX)

S3 Appendix Costs related to manual hiMSC and EV treatment manufacturing process. (DOCX)

S4 Appendix Price indices. (DOCX)

S5 Appendix Sensitivity analyses results. (DOCX)
