# Supplementary material for: HiMSC and EV derived treatments increase Quality of Life and reduce amount of Knee Replacement Surgeries compared to current standard of care for knee osteoarthritis patients in The Netherlands
Source: PLoS One. 2026 Mar 26;21(3):e0344203. doi: 10.1371/journal.pone.0344203 (PMC13020836; doi:10.1371/journal.pone.0344203)
Supplement: S1 Appendix — (DOCX) [file pone.0344203.s001.docx]

**S1 Appendix: Transition probabilities**

**Table 1**: Probability of knee replacement revision after total knee replacement (1)

| Year(s) since TKR | Probability of TKRR |
| --- | --- |
| 1 | 0.4 |
| 2 | 0.65 |
| 3 | 0.65 |
| 4 | 0.35 |
| 5 | 0.35 |
| 6* | 0.2 |

Abbreviations: TKR: total knee replacement; TKRR: total knee replacement revision. 
*: After six years the probability remains the same

**Table 2:** Background mortality per year (2)

| Age (December 31) | Probability of dying |
| --- | --- |
| 45 years | 0.00116 |
| 46 years | 0.00148 |
| 47 years | 0.00151 |
| 48 years | 0.00174 |
| 49 years | 0.00187 |
| 50 years | 0.00196 |
| 51 years | 0.00242 |
| 52 years | 0.00261 |
| 53 years | 0.00283 |
| 54 years | 0.00292 |
| 55 years | 0.00315 |
| 56 years | 0.00339 |
| 57 years | 0.00381 |
| 58 years | 0.0042 |
| 59 years | 0.0049 |
| 60 years | 0.00536 |
| 61 years | 0.00622 |
| 62 years | 0.00684 |
| 63 years | 0.00749 |
| 64 years | 0.00877 |
| 65 years | 0.00915 |
| 66 years | 0.01029 |
| 67 years | 0.01123 |
| 68 years | 0.01243 |
| 69 years | 0.01367 |
| 70 years | 0.01451 |
| 71 years | 0.01676 |
| 72 years | 0.01835 |
| 73 years | 0.02015 |
| 74 years | 0.02171 |
| 75 years | 0.02607 |
| 76 years | 0.0293 |
| 77 years | 0.03195 |
| 78 years | 0.03644 |
| 79 years | 0.04109 |
| 80 years | 0.04528 |
| 81 years | 0.05198 |
| 82 years | 0.05726 |
| 83 years | 0.06627 |
| 84 years | 0.07576 |

**References**

1. van der Woude JA, Nair SC, Custers RJ, van Laar JM, Kuchuck NO, Lafeber FP, et al. Knee Joint Distraction Compared to Total Knee Arthroplasty for Treatment of End Stage Osteoarthritis: Simulating Long-Term Outcomes and Cost-Effectiveness. PloS one. 2016;11(5):e0155524.

2. Unknown. Levensverwachting; geslacht, leeftijd (per jaar en periode van vijf jaren): CBS StatLine; 2024 [Available from: <https://opendata.cbs.nl/statline/#/CBS/nl/dataset/37360ned/table?fromstatweb>.
