# Supplementary material for: HiMSC and EV derived treatments increase Quality of Life and reduce amount of Knee Replacement Surgeries compared to current standard of care for knee osteoarthritis patients in The Netherlands
Source: PLoS One. 2026 Mar 26;21(3):e0344203. doi: 10.1371/journal.pone.0344203 (PMC13020836; doi:10.1371/journal.pone.0344203)
Supplement: S2 Appendix — (DOCX) [file pone.0344203.s002.docx]

**S2 Appendix: Costs related to automated hiMSC and EV treatment manufacturing process**

**Table 1**: Facility costs

| Item | Costs (€) |
| --- | --- |
| Facility Maintenance | 24,760 |
| Annual certification of Cleanrooms | 7,800 |
| Gowns and cleanroom garb | 24,000 |
| Calibrations | 16,619 |
| Disinfectants | 7,547 |
| QC Test equipment maintenance | 7,140 |
| Sterile gloves and masks | 6,342 |
| EM Plates, GP and IDs | 5,880 |
| Qualified Person | 32,177 |
| GMP HPRA INSPECTION (Inspector and QP Hours) | 16,800 |
| GMP Licence | 7,800 |
| Total | 156,864 |

Abbreviations: QC: quality control; EM: environmental monitoring; GP: growth promotion; ID: identification; GMP: good manufacturing practice; HPRA: health products regulatory authority.

**Table 2**: Staffing costs

| Position | Salary (€) |
| --- | --- |
| Management Production/QC | 200,000 |
| Production technologist (2x 0.5 FTE) | 66,282 |
| Automation Scientist (2x 0.5 FTE) | 66,282 |
| QC/Micro Scientist (2x 0.5 FTE) | 89,250 |
| Total | 421,814 |

Abbreviations: QC: quality control; FTE: full-time equivalent.

**Table 3**: Equipment costs

| *StemCell Discovery* | Depreciation costs (10 years) (€) |
| --- | --- |
| CoolGate (4°C) | 200 |
| Centrifuge | 1,968 |
| Plategate (RT) | 150 |
| Freezer (-20 °C) | 100 |
| PipetteGate | 300 |
| PlateSealer | 1,948 |
| Incubator | 18,600 |
| PCR | 4,174 |
| LH=45U | 15,281 |
| 96 pipetting head - UPGRADE CO-RE 96 1000ul II | 5,609 |
| Endotoxin Tester | 2,572 |
| Decapper 50 mL | 150 |
| Decapper 1-5 mL | 80 |
| High-Speed Microscope | 18,000 |
| Plate Reader (Spark cyto) | 12,000 |
| Frame | 1,000 |
| Cleanroom Housing | 1,660 |
| Gripper | 50 |
| Robotic Arm | 2,708 |
| PLC cabinet | 1,000 |
| *Autostem* |  |
| Frame | 1,158 |
| Cleanroom Housing | 15,152 |
| Centrifuge | 1,968 |
| Robotic arm | 2,708 |
| Gripper | 50 |
| Bioreactor (Eppendorf) | 5,406 |
| Bioreactor (Mobius) | 3,194 |
| Nucleocounter NC 3000 | 3,710 |
| Freezer (-80°C) | 340 |
| Sampling Station | 150 |
| Pump & Valve Station | 324 |
| Cassette/CoolContainer/Vial Gate | 300 |
| Hatch | 350 |
| Decapper | 400 |
| Pipette | 354 |
| PipetteGate | 200 |
| Vial/CentrifugeFlaskGate | 250 |
| Cleaning Equipment | 200 |
| Particle Counter | 701 |
| Cooler | 203 |
| PLC Cabinet | 5,000 |
| *EV Module* |  |
| Frame | 100 |
| Cleanroom Hatch | 150 |
| FPLC | 6,780 |
| Total | 136,697 |

Abbreviations: °C: degrees celcius; PCR: polymerase chain reaction; LHU: liquid handling unit; PLC: programmable logic controller; FPLC: fast protein liquid chromatography.

**Table 4**: Costs of consumables

| Process | No. of runs | Cost per run (€) | Total cost (€) |
| --- | --- | --- | --- |
| hiPSC cell bank | 1 | 7,198.00 | 7,198.00 |
| hiMSC differentiation and expansion to P3 | 3 | 11,841.00 | 35,524.00 |
| hMSC expansion in bioreactor (2 bioreactors run in parallel) | 3 | 12,148.00 | 36,444.00 |
| Production runs (2 bioreactors run in parallel) | 14 | 12,148.00 | 170,072.00 |
| Total |  |  | 249,238.00 |

Abbreviations: No: number; hiPSC: human-induced pluripotent stem cells; hiMSC: human-induced mesenchymal stromal cells; P3: passage 3.

**Table 5**: Quality control costs

| Process | No. of runs | Cost per run (€) | Total cost (€) |
| --- | --- | --- | --- |
| hiPSC cell bank | 1 | 5,000.00 | 5,000.00 |
| hiMSC differentiation and expansion to P3 | 3 | 5,000.00 | 15,000.00 |
| hiMSC expansion in bioreactor (2 bioreactors run in parallel) | 3 | 10,000.00 | 30,000.00 |
| Production runs (2 bioreactors run in parallel) | 14 | 10,000.00 | 140,000.00 |
| Total |  |  | 190,000.00 |

Abbreviations: No: number; hiPSC: human-induced pluripotent stem cells; hiMSC: human-induced mesenchymal stromal cells; P3: passage 3.
