# Supplementary material for: HiMSC and EV derived treatments increase Quality of Life and reduce amount of Knee Replacement Surgeries compared to current standard of care for knee osteoarthritis patients in The Netherlands
Source: PLoS One. 2026 Mar 26;21(3):e0344203. doi: 10.1371/journal.pone.0344203 (PMC13020836; doi:10.1371/journal.pone.0344203)
Supplement: S3 Appendix — (DOCX) [file pone.0344203.s003.docx]

**S3 Appendix: Costs related to manual hiMSC and EV treatment manufacturing process**

**Table 1**: Facility costs

| Item | Costs (€) |
| --- | --- |
| Facility Maintenance | 99,670.00 |
| Annual certification of Cleanrooms | 7,800.00 |
| Gowns and cleanroom garb | 24,000.00 |
| Calibrations | 16,619.00 |
| Disinfectants | 7,547.00 |
| QC Test equipment maintenance | 7,140.00 |
| Sterile gloves and masks | 6,342.00 |
| EM Plates, GP and IDs | 5,880.00 |
| Qualified Person | 32,176.00 |
| GMP HPRA INSPECTION (Inspector and QP Hours) | 16,800.00 |
| GMP Licence | 7,800.00 |
| Total | 231,775.00 |

Abbreviations: QC: quality control; EM: environmental monitoring; GP: growth promotion; ID: identification; GMP: good manufacturing practice; HPRA: health products regulatory authority.

**Table 2**: Staffing costs

| Position | Salary (€) |
| --- | --- |
| Management Production/QC | 200,000.00 |
| Production technologist (2x) | 132,564.00 |
| QC/Micro Scientist | 122,391.00 |
| Total | 421,814.00 |

Abbreviations: QC: quality control; FTE: full-time equivalent.

**Table 3**: Equipment costs

| *StemCell Discovery* | Depreciation costs (10 years) (€) |
| --- | --- |
| Centrifuge (x2) | 3,600.00 |
| Freezer (-20 °C) | 100.00 |
| Incubator | 1,100.00 |
| PCR | 4,175.00 |
| Endotoxin Tester | 2,572.00 |
| Microscope | 1,000.00 |
| Plate Reader | 12,000.00 |
| Bioreactor controller (X2) | 13,000.00 |
| Nucleocounter | 2,500.00 |
| FPLC | 6,800.00 |
| Laminar flow hoods (x2) | 2,400.00 |
| QC incubator | 400.00 |
| Total | 49,646.00 |

Abbreviations: °C: degrees celcius; PCR: polymerase chain reaction; FPLC: fast protein liquid chromatography.

**Table 4**: Costs of consumables

| Process | No. of runs | Cost per run (€) | Total cost (€) |
| --- | --- | --- | --- |
| hiPSC cell bank | 1 | 7,198.00 | 7,198.00 |
| hiMSC differentiation and expansion to P3 | 2 | 11,841.00 | 23,682.00 |
| hiMSC expansion in bioreactor (2 bioreactors run in parallel) | 2 | 12,148.00 | 24,296.00 |
| Production runs (2 bioreactors run in parallel) | 10 | 12,148.00 | 121,480.00 |
| Total |  |  | 176,656.00 |

Abbreviations: No: number; hiPSC: human-induced pluripotent stem cells; hiMSC: human-induced mesenchymal stromal cells; P3: passage 3. 

**Table 5**: Quality control costs

| Process | No. of runs | Cost per run (€) | Total cost (€) |
| --- | --- | --- | --- |
| hiPSC cell bank | 1 | 5,000.00 | 5,000.00 |
| hiMSC differentiation and expansion to P3 | 2 | 5,000.00 | 10,000.00 |
| hiMSC expansion in bioreactor (2 bioreactors run in parallel) | 2 | 10,000.00 | 20,000.00 |
| Production runs (2 bioreactors run in parallel) | 10 | 10,000.00 | 100,000.00 |
| Total |  |  | 135,000.00 |

Abbreviations: No: number; hiPSC: human-induced pluripotent stem cells; hiMSC: human-induced mesenchymal stromal cells; P3: passage 3.
