# Supplementary material for: HiMSC and EV derived treatments increase Quality of Life and reduce amount of Knee Replacement Surgeries compared to current standard of care for knee osteoarthritis patients in The Netherlands
Source: PLoS One. 2026 Mar 26;21(3):e0344203. doi: 10.1371/journal.pone.0344203 (PMC13020836; doi:10.1371/journal.pone.0344203)
Supplement: S4 Appendix — (DOCX) [file pone.0344203.s004.docx]

**S4 Appendix: Price indices**

**Table 1**: Price indices material and personnel costs (1)

| Year | Price indices material costs (%) | Price indices personnel costs (%) |
| --- | --- | --- |
| 2024 (provisional) | 2.51 | 4.94 |
| 2023 | 7.02 | 6.36 |
| 2022 | 9.25 | 4.42 |
| 2021 | 1.77 | 2.01 |
| 2020 | 1.97 | 3.28 |
| 2019 | 2.49 | 3.42 |
| 2018 | 1.55 | 2.96 |
| 2017 | 1.87 | 2.04 |
| 2016 | 0.38 | 1.74 |
| 2015 | 0.32 | 0.08 |
| 2014 | 1.04 | 1.94 |
| 2013 | 2.88 | 2.64 |
| 2012 | 2.41 | 2.95 |
| 2011 | 1.98 | 3.11 |
| 2010 | -0.31 |  |

**References**

1. Unknown. Prijsindexcijfers langdurige zorg: NZA; 2024 [Available from: <https://www.nza.nl/zorgsectoren/langdurige-zorg/veelgestelde-vragen/prijsindexcijfers>.
