# Supplementary material for: HiMSC and EV derived treatments increase Quality of Life and reduce amount of Knee Replacement Surgeries compared to current standard of care for knee osteoarthritis patients in The Netherlands
Source: PLoS One. 2026 Mar 26;21(3):e0344203. doi: 10.1371/journal.pone.0344203 (PMC13020836; doi:10.1371/journal.pone.0344203)
Supplement: S5 Appendix — (DOCX) [file pone.0344203.s005.docx]

**S5 Appendix: Sensitivity analyses results**

**Figure 1:** Top 10 model input parameters that are the main cost drivers for automatically produced hiMSC treatment from the hospital perspective (expected costs per patient over 40 years on the x-axis, parameters on the y-axis)


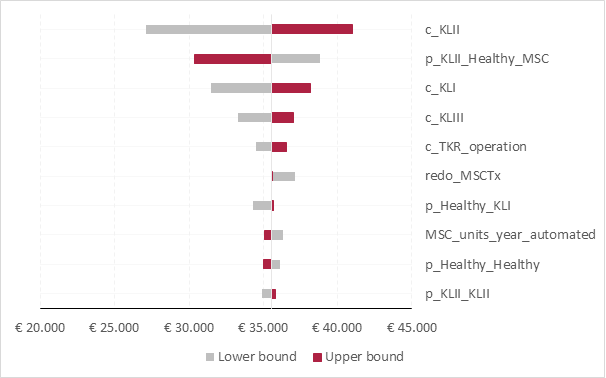
 Abbreviations: c_KLII: yearly costs of health state Kellgren-Lawrence II; p_KLII_Healthy_MSC: probability of regressing from KL state II to health state Healthy after receiving hiMSC treatment c_KLI: yearly costs of health state KL I; c_KLIII: yearly costs of health state KL III; c_TKR_operation: costs of TKR procedure; redo_MSCTx: possibility of receiving hiMSC treatment again when entering health state KL II; p_Healthy_KLI: probability of progressing to state KL I from health state Healthy; MSC_units_year_automated: the number of treatment units that can be produced per year; p_Healthy_Healthy: probability of staying in health state Healthy; p_KLII_KLII: probability of staying in health state KL II; KL: Kellgren-Lawrence; hiMSC: human induced mesenchymal stromal cells; TKR: total knee replacement; OA: osteoarthritis.

**Table 1:** Results of the sensitivity analysis of the top 10 model input parameters, that are the main cost-drivers for hiMSC treatment from the hospital perspective, when the cells are produced automatically

| **Parameter** | **Expected costs per patient over 40 years, lower bound (€)** | **Expected costs per patient over 40 years, upper bound (€)** | **Difference (€)** |
| --- | --- | --- | --- |
| *Baseline* | *35,569.25* | *35,569.25* |  |
| c_KLII | 27,075.46 | 40,974.41 | 13,898.95 |
| p_KLII_Healthy_MSC | 38,808.62 | 30,378.89 | -8,429.73 |
| c_KLI | 31,483.23 | 38,169.44 | 6,686.21 |
| c_KLIII | 33,283.02 | 37,024.12 | 3,741.10 |
| c_TKR_operation | 34,476.96 | 36,536.27 | 2,059.32 |
| redo_MSCTx | 37,164.59 | 35,569.25 | -1,595.34 |
| p_Healthy_KLI | 34,276.76 | 35,687.21 | 1,410.45 |
| MSC_units_year_automated | 36,347.11 | 35,050.68 | -1,296.42 |
| p_Healthy_Healthy | 36,123.47 | 35,046.86 | -1,076.61 |
| p_KLII_KLII | 34,894.09 | 35,789.66 | 895.57 |

Abbreviations: c_KLII: yearly costs of health state Kellgren-Lawrence II; p_KLII_Healthy_MSC: probability of regressing from KL state II to health state Healthy after receiving hiMSC treatment c_KLI: yearly costs of health state KL I; c_KLIII: yearly costs of health state KL III; c_TKR_operation: costs of TKR procedure; redo_MSCTx: possibility of receiving hiMSC treatment again when entering health state KL II; p_Healthy_KLI: probability of progressing to state KL I from health state Healthy; MSC_units_year_automated: the number of treatment units that can be produced per year; p_Healthy_Healthy: probability of staying in health state Healthy; p_KLII_KLII: probability of staying in health state KL II; KL: Kellgren-Lawrence; hiMSC: human induced mesenchymal stromal cells; TKR: total knee replacement; OA: osteoarthritis.

**Figure 2:** Top 10 model input parameters that are the main cost-drivers for automatically produced hiMSC treatment from the societal perspective (expected costs per patient over 40 years on the x-axis, parameters on the y-axis)


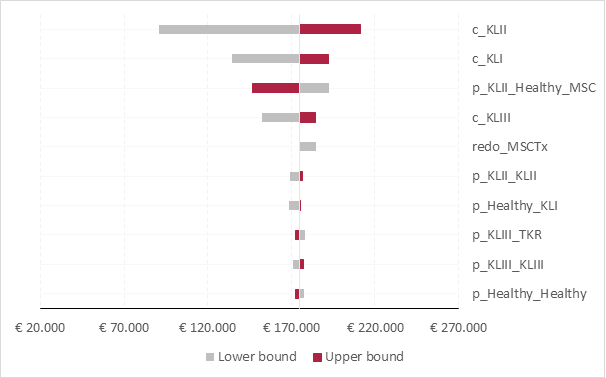
 Abbreviations: c_KLII: yearly costs of health state Kellgren-Lawrence II; c_KLI: yearly costs of health state KL I; p_KLII_Healthy_MSC: probability of regressing from KL state II to health state Healthy after receiving hiMSC treatment; c_KLIII: yearly costs of health state KL III; redo_MSCTx: possibility of receiving MSC treatment again when entering health state KL II; p_KLII_KLII: probability of staying in health state KL II; p_Healthy_KLI: probability of progressing to state KL I from health state Healthy; p_KLIII_TKR: probability of undergoing TKR from health state KL III; p_KLIII_KLIII: probability of staying in health state KL III; p_Healthy_Healthy: probability of staying in health state Healthy; hiMSC: human induced mesenchymal stromal cells; TKR: total knee replacement; OA: osteoarthritis.

**Table 2:** Results of the sensitivity analysis of the top 10 model input parameters that are the main cost drivers for hiMSC treatment from the societal perspective, when the cells are produced automatically

| **Parameter** | **Expected costs per patient over 40 years, lower bound (€)** | **Expected costs per patient over 40 years, upper bound (€)** | **Difference (€)** |
| --- | --- | --- | --- |
| *Baseline* | *175.023,42* | *175.023,42* |  |
| c_KLII | 90.747,15 | 211.315,17 | 120.568,02 |
| c_KLI | 134.481,65 | 192.481,83 | 58.000,18 |
| p_KLII_Healthy_MSC | 192.737,45 | 146.591,40 | -46.146,06 |
| c_KLIII | 152.339,29 | 184.791,85 | 32.452,56 |
| redo_MSCTx | 185.113,86 | 175.023,42 | -10.090,44 |
| p_KLII_KLII | 169.000,00 | 176.999,86 | 7.999,86 |
| p_Healthy_KLI | 168.486,48 | 175.619,97 | 7.133,50 |
| p_KLIII_TKR | 178.480,44 | 172.386,88 | -6.093,56 |
| p_KLIII_KLIII | 171.263,40 | 177.109,99 | 5.846,60 |
| p_Healthy_Healthy | 177.826,92 | 172.380,83 | -5.446,09 |

Abbreviations: hiMSC: human induced mesenchymal stromal cells; c_KLII: yearly costs of health state Kellgren-Lawrence II; c_KLI: yearly costs of health state KL I; p_KLII_Healthy_MSC: probability of regressing from KL state II to health state Healthy after receiving hiMSC treatment; c_KLIII: yearly costs of health state KL III; redo_MSCTx: possibility of receiving hiMSC treatment again when entering health state KL II; p_KLII_KLII: probability of staying in health state KL II; p_Healthy_KLI: probability of progressing to state KL I from health state Healthy; p_KLIII_TKR: probability of undergoing TKR from health state KL III; p_KLIII_KLIII: probability of staying in health state KL III; p_Healthy_Healthy: probability of staying in health state Healthy; KL: Kellgren-Lawrence; TKR: total knee replacement; OA: osteoarthritis.

**Figure 3:** Top 10 model input parameters that are the main cost-drivers for manually produced hiMSC treatment from the hospital perspective (expected costs per patient over 40 years on the x-axis, parameters on the y-axis)


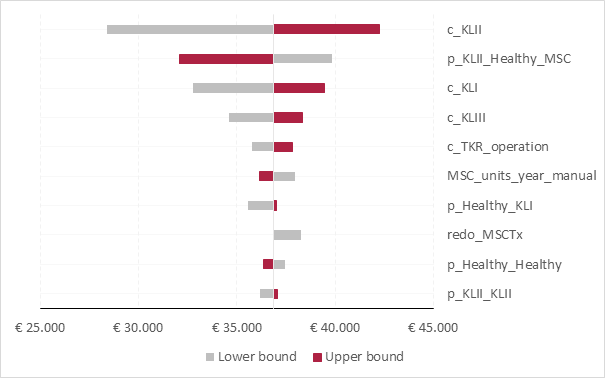
 Abbreviations: c_KLII: yearly costs of health state Kellgren-Lawrence II; p_KLII_Healthy_MSC: probability of regressing from KL state II to health state Healthy after receiving hiMSC treatment; c_KLI: yearly costs of health state KL I; c_KLIII: yearly costs of health state KL III; c_TKR_operation: costs of TKR procedure; MSC_units_year_manual: the number of treatment units that can be produced per year; p_Healthy_KLI: probability of progressing to state KL I from health state Healthy; redo_MSCTx: possibility of receiving hiMSC treatment again when entering health state KL II; p_Healthy_Healthy: probability of staying in health state Healthy; p_KLII_KLII: probability of staying in health state KL II; KL: Kellgren-Lawrence; hiMSC: human induced mesenchymal stromal cells; TKR: total knee replacement; OA: osteoarthritis.

**Table 3:** Result of the sensitivity analysis of the top 10 model input parameters that are the main cost-drivers for hiMSC treatment from the hospital perspective, when the cells are manually produced

| **Parameter** | **Expected costs per patient over 40 years, lower bound (€)** | **Expected costs per patient over 40 years, upper bound (€)** | **Difference (€)** |
| --- | --- | --- | --- |
| *Baseline* | *36,870.66* | *36,870.66* |  |
| c_KLII | 28,376.86 | 42,275.81 | 13,898.95 |
| p_KLII_Healthy_MSC | 39,853.73 | 32,062.52 | -7,791.21 |
| c_KLI | 32,784.63 | 39,470.85 | 6,686.21 |
| c_KLIII | 34,584.43 | 38,325.53 | 3,741.10 |
| c_TKR_operation | 35,778.36 | 37,837.68 | 2,059.32 |
| MSC_units_year_manual | 37,973.86 | 36,135.19 | -1,838.67 |
| p_Healthy_KLI | 35,540.75 | 36,992.00 | 1,451.25 |
| redo_MSCTx | 38,253.47 | 36,870.66 | -1,382.81 |
| p_Healthy_Healthy | 37,440.41 | 36,333.53 | -1,106.88 |
| p_KLII_KLII | 36,195.49 | 37,091.07 | 895.57 |

Abbreviations: c_KLII: yearly costs of health state Kellgren-Lawrence II; p_KLII_Healthy_MSC: probability of regressing from KL state II to health state Healthy after receiving hiMSC treatment; c_KLI: yearly costs of health state KL I; c_KLIII: yearly costs of health state KL III; c_TKR_operation: costs of TKR procedure; MSC_units_year_manual: the number of treatment units that can be produced per year; p_Healthy_KLI: probability of progressing to state KL I from health state Healthy; redo_MSCTx: possibility of receiving hiMSC treatment again when entering health state KL II; p_Healthy_Healthy: probability of staying in health state Healthy; p_KLII_KLII: probability of staying in health state KL II; KL: Kellgren-Lawrence; hiMSC: human-induced mesenchymal stromal cells; TKR: total knee replacement; OA: osteoarthritis.

**Figure 4:** Top 10 model input parameters that are the main cost-drivers for manually produced hiMSC treatment from the societal perspective (expected costs per patient over 40 years on the x-axis, parameters on the y-axis)


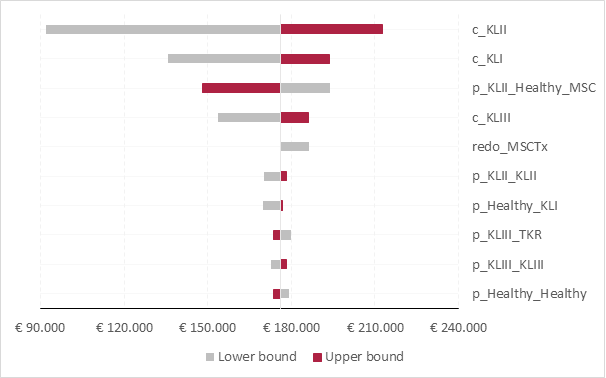
 Abbreviations: c_KLII: yearly costs of health state Kellgren-Lawrence II; c_KLI: yearly costs of health state KL I; p_KLII_Healthy_MSC: probability of regressing from KL state II to health state Healthy after receiving hiMSC treatment; c_KLIII: yearly costs of health state KL III; redo_MSCTx: possibility of receiving hiMSC treatment again when entering health state KL II; p_KLII_KLII: probability of staying in health state KL II; p_Healthy_KLI: probability of progressing to state KL I from health state Healthy; p_KLIII_TKR: probability of undergoing TKR from health state KL III; p_KLIII_KLIII: probability of staying in health state KL III; p_Healthy_Healthy: probability of staying in health state Healthy; KL: Kellgren-Lawrence; hiMSC: human induced mesenchymal stromal cells; TKR: total knee replacement; OA: osteoarthritis.

**Table 4:** Results of the sensitivity analysis of the top 10 model input parameters that are the main cost-drivers for hiMSC treatment from the societal perspective, when the cells are manually produced

| **Parameter** | **Expected costs per patient over 40 years, lower bound (€)** | **Expected costs per patient over 40 years, upper bound (€)** | **Difference (€)** |
| --- | --- | --- | --- |
| *Baseline* | *176.324,82* | *176.324,82* |  |
| c_KLII | 92.048,55 | 212.616,58 | 120.568,02 |
| c_KLI | 135.783,05 | 193.783,24 | 58.000,18 |
| p_KLII_Healthy_MSC | 193.782,56 | 148.275,03 | -45.507,54 |
| c_KLIII | 153.640,69 | 186.093,25 | 32.452,56 |
| redo_MSCTx | 186.202,75 | 176.324,82 | -9.877,92 |
| p_KLII_KLII | 170.301,40 | 178.301,26 | 7.999,86 |
| p_Healthy_KLI | 169.750,47 | 176.924,77 | 7.174,30 |
| p_KLIII_TKR | 179.781,85 | 173.688,28 | -6.093,56 |
| p_KLIII_KLIII | 172.564,80 | 178.411,40 | 5.846,60 |
| p_Healthy_Healthy | 179.143,86 | 173.667,50 | -5.476,36 |

Abbreviations: hiMSC: human induced mesenchymal stromal cells; c_KLII: yearly costs of health state Kellgren-Lawrence II; c_KLI: yearly costs of health state KL I; p_KLII_Healthy_MSC: probability of regressing from KL state II to health state Healthy after receiving hiMSC treatment; c_KLIII: yearly costs of health state KL III; redo_MSCTx: possibility of receiving hiMSC treatment again when entering health state KL II; p_KLII_KLII: probability of staying in health state KL II; p_Healthy_KLI: probability of progressing to state KL I from health state Healthy; p_KLIII_TKR: probability of undergoing TKR from health state KL III; p_KLIII_KLIII: probability of staying in health state KL III; p_Healthy_Healthy: probability of staying in health state Healthy; KL: Kellgren-Lawrence; TKR: total knee replacement; OA: osteoarthritis.

**Figure 5:** Top 10 model input parameters that are the main cost drivers for automatically produced hiMSC and EV treatments from the hospital perspective (expected costs per patient over 40 years on the x-axis, parameters on the y-axis)


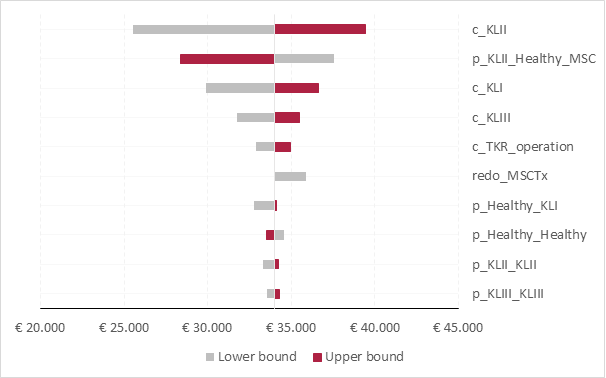
 Abbreviations: c_KLII: yearly costs of health state Kellgren-Lawrence II; p_KLII_Healthy_MSC: probability of regressing from KL state II to health state Healthy after receiving hiMSC or EV treatment; c_KLI: yearly costs of health state KL I; c_KLIII: yearly costs of health state KL III; c_TKR_operation: costs of TKR procedure; redo_MSCTx: possibility of receiving hiMSC or EV treatment again when entering health state KL II; p_Healthy_KLI: probability of progressing to state KL I from health state Healthy; p_Healthy_Healthy: probability of staying in health state Healthy; p_KLII_KLII: probability of staying in health state KL II; p_KLIII_KLIII: probability of staying in health state KL III; KL: Kellgren-Lawrence; hiMSC: human induced mesenchymal stromal cells; EV: extracellular vesicles; TKR: total knee replacement; OA: osteoarthritis.

**Table 5:** Results of the sensitivity analysis of the top 10 model input parameters that are the main cost-drivers for hiMSC and EV treatments from the hospital perspective, when the cells are automatically produced

| **Parameter** | **Expected costs per patient over 40 years, lower bound (€)** | **Expected costs per patient over 40 years, upper bound (€)** | **Difference (€)** |
| --- | --- | --- | --- |
| *Baseline* | *34,013.54* | *34,013.54* |  |
| c_KLII | 25,519.75 | 39,418.70 | 13,898.95 |
| p_KLII_Healthy_MSC | 37,559.29 | 28,366.27 | -9,193.03 |
| c_KLI | 29,927.52 | 36,613.73 | 6,686.21 |
| c_KLIII | 31,727.32 | 35,468.42 | 3,741.10 |
| c_TKR_operation | 32,921.25 | 34,980.56 | 2,059.32 |
| redo_MSCTx | 35,862.93 | 34,013.54 | -1,849.38 |
| p_Healthy_KLI | 32,765.77 | 34,127.44 | 1,361.67 |
| p_Healthy_Healthy | 34,549.18 | 33,508.76 | -1,040.41 |
| p_KLII_KLII | 33,338.38 | 34,233.96 | 895.57 |
| p_KLIII_KLIII | 33,537.14 | 34,274.17 | 737.03 |

Abbreviations: c_KLII: yearly costs of health state Kellgren-Lawrence II; p_KLII_Healthy_MSC: probability of regressing from KL state II to health state Healthy after receiving hiMSC or EV treatment; c_KLI: yearly costs of health state KL I; c_KLIII: yearly costs of health state KL III; c_TKR_operation: costs of TKR procedure; redo_MSCTx: possibility of receiving hiMSC or EV treatment again when entering health state KL II; p_Healthy_KLI: probability of progressing to state KL I from health state Healthy; p_Healthy_Healthy: probability of staying in health state Healthy; p_KLII_KLII: probability of staying in health state KL II; p_KLIII_KLIII: probability of staying in health state KL III; KL: Kellgren-Lawrence; hiMSC: human induced mesenchymal stromal cells; EV: extracellular vesicles; TKR: total knee replacement; OA: osteoarthritis.

**Figure 6:** Top 10 model input parameters that are the main cost-drivers for automatically produced hiMSC and EV treatments from the societal perspective (expected costs per patient over 40 years on the x-axis, parameters on the y-axis)


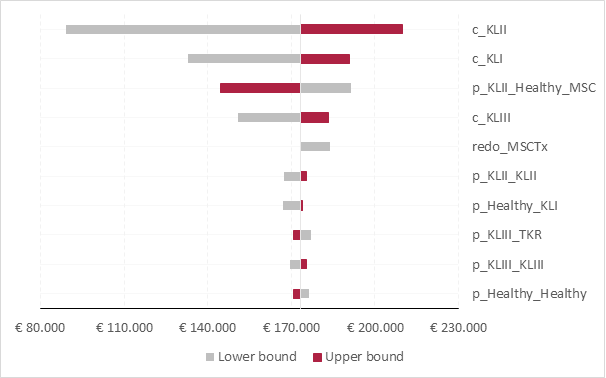
 Abbreviations: c_KLII: yearly costs of health state Kellgren-Lawrence II; c_KLI: yearly costs of health state KL I; p_KLII_Healthy_MSC: probability of regressing from KL state II to health state Healthy after receiving hiMSC or EV treatment; c_KLIII: yearly costs of health state KL III; redo_MSCTx: possibility of receiving hiMSC or EV treatment again when entering health state KL II; p_KLII_KLII: probability of staying in health state KL II; p_Healthy_KLI: probability of progressing to state KL I from health state Healthy; p_KLIII_TKR: probability of undergoing TKR from health state KL III; p_KLIII_KLIII: probability of staying in health state KL III; p_Healthy_Healthy: probability of staying in health state Healthy; KL: Kellgren-Lawrence; hiMSC: human induced mesenchymal stromal cells; EV: extracellular vesicles; TKR: total knee replacement; OA: osteoarthritis.

**Table 6:** Results of the sensitivity analysis of the top 10 model input parameters that are the main cost drivers for hiMSC and EV treatments from the societal perspective, when the cells are automatically produced

| **Parameter** | **Expected costs per patient over 40 years, lower bound (€)** | **Expected costs per patient over 40 years, upper bound (€)** | **Difference (€)** |
| --- | --- | --- | --- |
| *Baseline* | *173.467,71* | *173.467,71* |  |
| c_KLII | 89.191,44 | 209.759,46 | 120.568,02 |
| c_KLI | 132.925,94 | 190.926,12 | 58.000,18 |
| p_KLII_Healthy_MSC | 191.488,12 | 144.578,78 | -46.909,35 |
| c_KLIII | 150.783,58 | 183.236,14 | 32.452,56 |
| redo_MSCTx | 183.812,20 | 173.467,71 | -10.344,49 |
| p_KLII_KLII | 167.444,29 | 175.444,15 | 7.999,86 |
| p_Healthy_KLI | 166.975,49 | 174.060,21 | 7.084,72 |
| p_KLIII_TKR | 176.924,74 | 170.831,17 | -6.093,56 |
| p_KLIII_KLIII | 169.707,69 | 175.554,29 | 5.846,60 |
| p_Healthy_Healthy | 176.252,63 | 170.842,73 | -5.409,90 |

Abbreviations: c_KLII: yearly costs of health state Kellgren-Lawrence II; c_KLI: yearly costs of health state KL I; p_KLII_Healthy_MSC: probability of regressing from KL state II to health state Healthy after receiving hiMSC or EV treatment; c_KLIII: yearly costs of health state KL III; redo_MSCTx: possibility of receiving hiMSC or EV treatment again when entering health state KL II; p_KLII_KLII: probability of staying in health state KL II; p_Healthy_KLI: probability of progressing to state KL I from health state Healthy; p_KLIII_TKR: probability of undergoing TKR from health state KL III; p_KLIII_KLIII: probability of staying in health state KL III; p_Healthy_Healthy: probability of staying in health state Healthy; KL: Kellgren-Lawrence; hiMSC: human induced mesenchymal stromal cells; EV: extracellular vesicles; TKR: total knee replacement; OA: osteoarthritis.

**Figure 7:** Top 10 model input parameters that are the main cost drivers for manually produced hiMSC and EV treatments from the hospital perspective (expected costs per patient over 40 years on the x-axis, parameters on the y-axis)


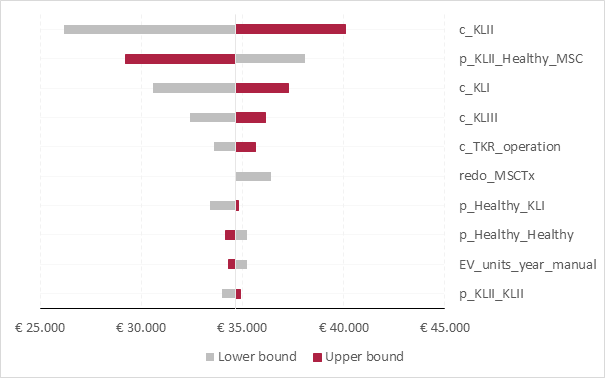
 Abbreviations: c_KLII: yearly costs of health state Kellgren-Lawrence II; p_KLII_Healthy_MSC: probability of regressing from KL state II to health state Healthy after receiving hiMSC or EV treatment; c_KLI: yearly costs of health state KL I; c_KLIII: yearly costs of health state KL III; c_TKR_operation: costs of TKR procedure; redo_MSCTx: possibility of receiving hiMSC or EV treatment again when entering health state KL II; p_Healthy_KLI: probability of progressing to state KL I from health state Healthy; p_Healthy_Healthy: probability of staying in health state Healthy; EV_units_year_manual: the number of treatment units that can be produced per year; p_KLII_KLII: probability of staying in health state KL II; KL: Kellgren-Lawrence; hiMSC: human induced mesenchymal stromal cells; EV: extracellular vesicles; TKR: total knee replacement; OA: osteoarthritis.

**Table 7:** Results of the sensitivity analysis of the top 10 model input parameters that are the main cost drivers for hiMSC and EV treatments from the hospital perspective, when the cells are manually produced

| **Parameter** | **Expected costs per patient over 40 years, lower bound (€)** | **Expected costs per patient over 40 years, upper bound (€)** | **Difference (€)** |
| --- | --- | --- | --- |
| *Baseline* | *34,664.25* | *34,664.25* |  |
| c_KLII | 26,170.45 | 40,069.40 | 13,898.95 |
| p_KLII_Healthy_MSC | 38,081.85 | 29,208.08 | -8,873.77 |
| c_KLI | 30,578.22 | 37,264.44 | 6,686.21 |
| c_KLIII | 32,378.02 | 36,119.12 | 3,741.10 |
| c_TKR_operation | 33,571.95 | 35,631.27 | 2,059.32 |
| redo_MSCTx | 36,407.37 | 34,664.25 | -1,743.12 |
| p_Healthy_KLI | 33,397.77 | 34,779.84 | 1,382.07 |
| p_Healthy_Healthy | 35,207.65 | 34,152.10 | -1,055.55 |
| EV_units_year_manual | 35,215.85 | 34,296.51 | -919.34 |
| p_KLII_KLII | 33,989.08 | 34,884.66 | 895.57 |

Abbreviations: c_KLII: yearly costs of health state Kellgren-Lawrence II; p_KLII_Healthy_MSC: probability of regressing from KL state II to health state Healthy after receiving hiMSC or EV treatment; c_KLI: yearly costs of health state KL I; c_KLIII: yearly costs of health state KL III; c_TKR_operation: costs of TKR procedure; redo_MSCTx: possibility of receiving hiMSC or EV treatment again when entering health state KL II; p_Healthy_KLI: probability of progressing to state KL I from health state Healthy; p_Healthy_Healthy: probability of staying in health state Healthy; EV_units_year_manual: the number of treatment units that can be produced per year; p_KLII_KLII: probability of staying in health state KL II; KL: Kellgren-Lawrence; hiMSC: human induced mesenchymal stromal cells; EV: extracellular vesicles; TKR: total knee replacement; OA: osteoarthritis.

**Figure 8:** Top 10 model input parameters that are the main cost drivers for manually produced hiMSC and EV treatments from the societal perspective (expected costs per patient over 40 years on the x-axis, parameters on the y-axis)


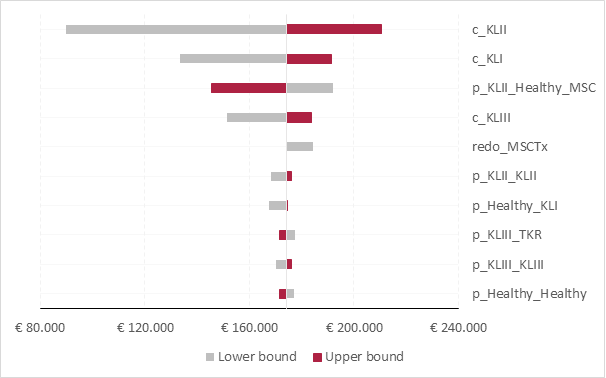
 Abbreviations: c_KLII: yearly costs of health state Kellgren-Lawrence II; c_KLI: yearly costs of health state KL I; p_KLII_Healthy_MSC: probability of regressing from KL state II to health state Healthy after receiving hiMSC or EV treatment; c_KLIII: yearly costs of health state KL III; redo_MSCTx: possibility of receiving hiMSC or EV treatment again when entering health state KL II; p_KLII_KLII: probability of staying in health state KL II; p_Healthy_KLI: probability of progressing to state KL I from health state Healthy; p_KLIII_TKR: probability of undergoing TKR from health state KL III; p_KLIII_KLIII: probability of staying in health state KL III; p_Healthy_Healthy: probability of staying in health state Healthy; KL: Kellgren-Lawrence; EV: extracellular vesicles; TKR: total knee replacement; OA: osteoarthritis.

**Table 8:** Results of the sensitivity analysis of the top 10 model input parameters that are the main cost drivers for hiMSC and EV treatments from the societal perspictive, when the cells are manually produced

| **Parameter** | **Expected costs per patient over 40 years, lower bound (€)** | **Expected costs per patient over 40 years, upper bound (€)** | **Difference (€)** |
| --- | --- | --- | --- |
| *Baseline* | *174.118,41* | *174.118,41* |  |
| c_KLII | 89.842,14 | 210.410,17 | 120.568,02 |
| c_KLI | 133.576,64 | 191.576,83 | 58.000,18 |
| p_KLII_Healthy_MSC | 192.010,68 | 145.420,59 | -46.590,09 |
| c_KLIII | 151.434,28 | 183.886,84 | 32.452,56 |
| redo_MSCTx | 184.356,65 | 174.118,41 | -10.238,23 |
| p_KLII_KLII | 168.094,99 | 176.094,85 | 7.999,86 |
| p_Healthy_KLI | 167.607,48 | 174.712,61 | 7.105,12 |
| p_KLIII_TKR | 177.575,44 | 171.481,87 | -6.093,56 |
| p_KLIII_KLIII | 170.358,39 | 176.204,99 | 5.846,60 |
| p_Healthy_Healthy | 176.911,10 | 171.486,07 | -5.425,03 |

Abbreviations: c_KLII: yearly costs of health state Kellgren-Lawrence II; c_KLI: yearly costs of health state KL I; p_KLII_Healthy_MSC: probability of regressing from KL state II to health state Healthy after receiving hiMSC or EV treatment; c_KLIII: yearly costs of health state KL III; redo_MSCTx: possibility of receiving hiMSC or EV treatment again when entering health state KL II; p_KLII_KLII: probability of staying in health state KL II; p_Healthy_KLI: probability of progressing to state KL I from health state Healthy; p_KLIII_TKR: probability of undergoing TKR from health state KL III; p_KLIII_KLIII: probability of staying in health state KL III; p_Healthy_Healthy: probability of staying in health state Healthy; KL: Kellgren-Lawrence; EV: extracellular vesicles; TKR: total knee replacement; OA: osteoarthritis.

**Figure 9:** Top 10 model input parameters that are the main cost drivers for standard of care from the hospital perspective (expected costs per patient over 40 years on the x-axis, parameters on the y-axis)


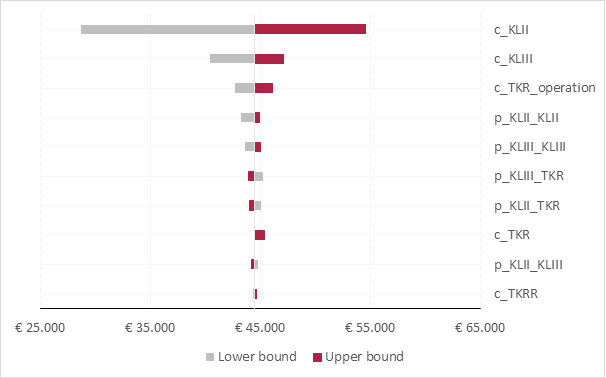
 Abbreviations: c_KLII: yearly costs of health state Kellgren-Lawrence II; c_KLIII: yearly costs of health state KL III; c_TKR_operation: costs of TKR procedure; p_KLII_KLII: probability of staying in health state KL II; p_KLIII_KLIII: probability of staying in health state KL III; p_KLIII_TKR: probability of undergoing TKR from health state KL III; p_KLII_TKR: probability of undergoing TKR from health state KL II; c_TKR: yearly costs of health state TKR; p_KLII_KLIII: probability of progressing from health state KL II to health state KL III; c_TKRR: yearly costs of health state TKRR; KL: Kellgren-Lawrence; TKR: total knee replacement; TKRR: total knee replacement revision; OA: osteoarthritis.

**Table 9**: Results of the sensitivity analysis of the top 10 model input parameters that are the main cost drivers for standard of care from the hospital perspective

| **Parameter** | **Expected costs per patient over 40 years, lower bound (€)** | **Expected costs per patient over 40 years, upper bound (€)** | **Difference (€)** |
| --- | --- | --- | --- |
| *Baseline* | *44,482.61* | *44,482.61* |  |
| c_KLII | 28,650.16 | 54,557.82 | 25,907.65 |
| c_KLIII | 40,389.55 | 47,087.28 | 6,697.73 |
| c_TKR_operation | 42,653.95 | 46,101.53 | 3,447.58 |
| p_KLII_KLII | 43,265.52 | 44,881.91 | 1,616.39 |
| p_KLIII_KLIII | 43,573.96 | 44,986.22 | 1,412.26 |
| p_KLIII_TKR | 45,262.82 | 43,883.60 | -1,379.22 |
| p_KLII_TKR | 45,064.80 | 44,012.85 | -1,051.95 |
| c_TKR | 44,482.61 | 45,381.28 | 898.67 |
| p_KLII_KLIII | 44,796.11 | 44,183.81 | -612.30 |
| c_TKRR | 44,356.90 | 44,668.55 | 311.65 |

Abbreviations: c_KLII: yearly costs of health state Kellgren-Lawrence II; c_KLIII: yearly costs of health state KL III; c_TKR_operation: costs of TKR procedure; p_KLII_KLII: probability of staying in health state KL II; p_KLIII_KLIII: probability of staying in health state KL III; p_KLIII_TKR: probability of undergoing TKR from health state KL III; p_KLII_TKR: probability of undergoing TKR from health state KL II; c_TKR: yearly costs of health state TKR; p_KLII_KLIII: probability of progressing from health state KL II to health state KL III; c_TKRR: yearly costs of health state TKRR; KL: Kellgren-Lawrence; TKR: total knee replacement; TKRR: total knee replacement revision; OA: osteoarthritis.

**Figure 10:** Top 10 model input parameters that are the main cost drivers for standard of care from the societal perspective (expected costs per patient over 40 years on the x-axis, parameters on the y-axis)


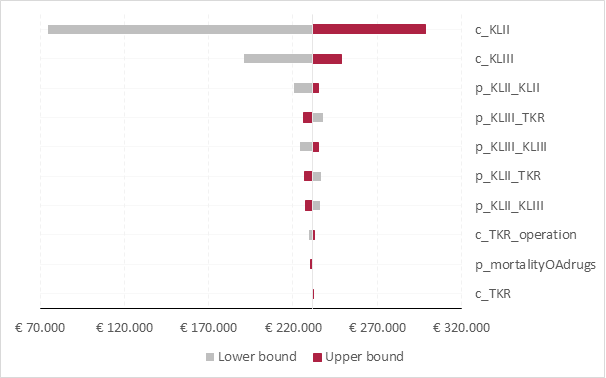
 Abbreviations: c_KLII: yearly costs of health state Kellgren-Lawrence II; c_KLIII: yearly costs of health state KL III; p_KLII_KLII: probability of staying in health state KL II; p_KLIII_TKR: probability of undergoing TKR from health state KL III; p_KLIII_KLIII: probability of staying in health state KL III; p_KLII_TKR: probability of undergoing TKR from health state KL II; p_KLII_KLIII: probability of progressing from health state KL II to health state KL III; c_TKR_operation: costs of TKR procedure; p_mortalityOAdrugs: probability of dying due to the use of OA drugs; c_TKR: yearly costs of health state TKR; KL: Kellgren-Lawrence; TKR: total knee replacement; OA: osteoarthritis.

**Table 10**: Results of the sensitivity analysis of the top 10 model input parameters that are the main cost-drivers for standard of care from the societal perspective

| **Parameter** | **Expected costs per patient over 40 years, lower bound (€)** | **Expected costs per patient over 40 years, upper bound (€)** | **Difference (€)** |
| --- | --- | --- | --- |
| *Baseline* | *231.382,41* | *231.382,41* |  |
| c_KLII | 74.291,34 | 299.030,27 | 224.738,94 |
| c_KLIII | 190.770,76 | 248.870,94 | 58.100,18 |
| p_KLII_KLII | 220.679,19 | 234.910,44 | 14.231,26 |
| p_KLIII_TKR | 238.085,43 | 226.348,82 | -11.736,61 |
| p_KLIII_KLIII | 224.396,07 | 235.299,72 | 10.903,65 |
| p_KLII_TKR | 236.658,67 | 227.148,11 | -9.510,57 |
| p_KLII_KLIII | 235.960,70 | 227.280,17 | -8.680,53 |
| c_TKR_operation | 229.553,75 | 233.001,33 | 3.447,58 |
| p_mortalityOAdrugs | 232.167,76 | 230.601,81 | -1.565,94 |
| c_TKR | 231.382,41 | 232.281,08 | 898,67 |

Abbreviations: c_KLII: yearly costs of health state Kellgren-Lawrence II; c_KLIII: yearly costs of health state KL III; p_KLII_KLII: probability of staying in health state KL II; p_KLIII_TKR: probability of undergoing TKR from health state KL III; p_KLIII_KLIII: probability of staying in health state KL III; p_KLII_TKR: probability of undergoing TKR from health state KL II; p_KLII_KLIII: probability of progressing from health state KL II to health state KL III; c_TKR_operation: costs of TKR procedure; p_mortalityOAdrugs: probability of dying due to the use of OA drugs; c_TKR: yearly costs of health state TKR; KL: Kellgren-Lawrence; TKR: total knee replacement; OA: osteoarthritis.
